# Clinical treatment considerations in the intensity-modulated radiotherapy era for patients with N0-category nasopharyngeal carcinoma and enlarged neck lymph nodes

**DOI:** 10.1186/s40880-017-0199-2

**Published:** 2017-03-24

**Authors:** Hao Peng, Lei Chen, Rui Guo, Yuan Zhang, Wen-Fei Li, Yan-Ping Mao, Ying Sun, Fan Zhang, Li-Zhi Liu, Li Tian, Jun Ma

**Affiliations:** 1 State Key Laboratory of Oncology in South China, Collaborative Innovation Center for Cancer Medicine, Sun Yat-sen University Cancer Center, Guangzhou, 510060 Guangdong P. R. China; 2 Department of Radiation Oncology, Sun Yat-sen University Cancer Center, Guangzhou, 510060 Guangdong P. R. China; 3 Imaging Diagnosis and Interventional Center, Sun Yat-sen University Cancer Center, Guangzhou, 510060 Guangdong P. R. China; 4 State Key Laboratory of Oncology in South China, Department of Radiation Oncology, Sun Yat-sen University Cancer Center, 651 Dongfeng Road East, Guangzhou, 510060 P. R. China

**Keywords:** Nasopharyngeal carcinoma, N0-category, Enlarged neck lymph node, Biological equivalent dose, Intensity-modulated radiotherapy, Prognosis

## Abstract

**Background:**

Nasopharyngeal carcinoma (NPC) shows a high proportion of lymph node metastasis, and treatment guidelines have been developed for positive nodes. However, no irradiation guidelines have been proposed for patients with enlarged neck lymph nodes (ENLNs) that do not meet the radiological criteria of 10 mm in diameter for positive lymph nodes. This study aimed to determine the prognostic value and radiation dose for ENLNs in N0-category NPC patients treated with intensity-modulated radiotherapy (IMRT).

**Methods:**

We reviewed the medical data of 251 patients with non-metastatic, N0-category NPC treated with IMRT. Receiver operating characteristic curves were used to calculate the cut-off value of the ENLN diameter for the prediction of disease failure. The biological equivalent dose (BED) for ENLNs was calculated. Patient survival was compared between the small and large ENLN groups. Independent prognostic factors were identified using the Cox proportional hazards model.

**Results:**

The estimated 4-year regional relapse-free survival rate was higher in patients with ENLNs ≥5.5 mm than in those with ENLNs <5.5 mm (100% vs. 98.8%, *P* = 0.049), whereas disease-free, overall, and distant metastasis-free survival rates were similar between the two groups. After adjusting for various factors, ENLN diameter was not identified as an independent prognostic factor (*P* > 0.05 for all survival rates). In the subgroup analysis, patients receiving BED ≥72 Gy had a similar prognosis as patients receiving BED <72 Gy in both the small and large ENLN groups. The multivariate analysis also confirmed that BED ≥72 Gy was not associated with significantly improved prognosis in patients with N0-category NPC.

**Conclusions:**

A BED of 72 Gy to ENLNs is considerably sufficient to provide a clinical benefit to patients with N0-category NPC. Prospective studies are warranted to validate the findings in the present study.

## Background

Nasopharyngeal carcinoma (NPC), a cancer arising from the nasopharyngeal epithelium, has an extremely unbalanced geographical distribution with an age-standardized incidence of 20–50 per 100,000 males in South China compared to 0.5 per 100,000 in predominantly white populations of European origin [1]. Due to the difficulty of radical resection and the high degree of radiosensitivity of NPC, radiotherapy has been the primary and only curative treatment for non-disseminated NPC. Chemotherapy has also been reported to provide a survival benefit to patients with advanced NPC [2]. For patients with lymph node (LN)-positive NPC, concurrent chemoradiotherapy (CCRT) alone or combined with neoadjuvant/adjuvant chemotherapy has been recommended as the standard treatment according to the National Comprehensive Cancer Network (NCCN) clinical practice guidelines [3].

NPC has a high proportion of LN metastasis (86.4%) [4], and the NCCN guidelines recommend a prescribed dose of 66–70 Gy to treat positive LNs. However, no irradiation guidelines have been proposed for patients with enlarged neck LNs (ENLNs) that do not meet the radiological criteria of 10 mm in diameter for positive LNs [5]. Indeed, it is difficult to assess the nature of such ENLNs because NPC is typically treated using radiotherapy without performing cervical nodal biopsies. In clinical practice, this issue remains controversial as many clinicians concern that these ENLNs may be positive and adversely affect prognosis. However, other clinicians have considered ENLNs to be negative and treat these tumors with a low prescribed radiation dose. This situation urgently needs to be addressed.

To the best of our knowledge, no studies to date have investigated the prognostic value of ENLNs in patients with N0-category NPC treated with intensity-modulated radiotherapy (IMRT). Therefore, we conducted a retrospective study to characterize this issue and provide clinical evidence to help clinicians guide the treatment of NPC in such situations.

## Methods

### Ethical agreement

The present study was approved by the Research Ethics Committee of Sun Yat-sen University Cancer Center (Guangzhou, China), and informed consent was obtained from all patients for the use of their data in clinical researches. The present study was conducted in accordance with the ethical standards of the institution.

### Patient selection

The data of patients with newly-diagnosed, non-disseminated NPC treated between November 2009 and February 2012 at Sun Yat-sen University Cancer Center were reviewed. Pre-treatment magnetic resonance imaging (MRI) data on the nasopharyngeal and cervical regions were thoroughly analyzed. The eligibility criteria were as follows: (1) N0-category disease; (2) World Health Organization (WHO) pathologic type II/III; and (3) age of 18 years or older.

### Clinical staging

The routine staging process included a complete history collection and clinical examination of the head and neck region, direct fiberoptic nasopharyngoscopy, MRI scans of the skull base, neck and chest radiography, a whole-body bone scan, and abdominal sonography. Positron emission tomography (PET)-computed tomography (CT) scans were also performed when clinically indicated. All patients received a dental evaluation before radiotherapy.

All cases were restaged according to the 7th edition of the International Union against Cancer/American Joint Committee on Cancer (UICC/AJCC) system [6]. All MRI materials and clinical records were reviewed to minimize heterogeneity in restaging. Two radiologists (L.Z.L and L.T) separately evaluated all the scans, and disagreements were resolved by consensus.

### MRI protocol

All patients underwent MRI using a 3-Tesla system (Trio Tim; Siemens, Erlangen, Germany). The region from the suprasellar cistern to the inferior margin at the sternal end of the clavicle was examined using a head-and-neck coil. T1-weighted fast spin-echo images on the axial, coronal, and sagittal planes (repetition time [TR]/echo time [TE], 650/9 ms), T2-weighted fast spin-echo images on the axial plane (TR/TE, 2470/90 ms), and a spin-echo echo-planar DWI sequence (matrix, 192 × 192; TR/TE, 5100/96 ms; *b* values, 0 and 1000 s/mm^2^; three signal averages) were obtained before contrast injection. After the intravenous administration of gadopentetate dimeglumine (0.1 mmol/kg body weight; Magnevist, Schering, Berlin, Germany), axial and sagittal T1-weighted spin-echo sequences and coronal T1-weighted fat-suppressed spin-echo sequences were performed sequentially using the same parameters as applied before the injection of gadopentetate dimeglumine. Subsequently, 5-mm-thick sections were obtained with a 1-mm interslice gap for the axial plane, and 6-mm-thick sections with a 1-mm interslice gap were obtained for the coronal and sagittal planes, resulting in a matrix size of 512 × 512.

### Measurement of the short diameter of the ENLNs

T2-weighted axial MRI data without contrast enhancement were reviewed. The short diameters of the ENLNs were measured separately by two radiologists with experience of more than 10 years. Disagreements were resolved by consensus. For patients with two or more ENLNs, the short diameter of the largest ENLN was measured. The maximum short diameter should be ≤10 mm for ENLNs of level IIa and ≤9 mm for ENLNs of other levels.

### Clinical treatment

#### Radiotherapy

All patients underwent IMRT at Sun Yat-sen University Cancer Center. Immobilization was achieved using a custom-made head-to-neck thermoplastic cast with the patient’s neck resting on a support. High-resolution planning CT scans with contrast were taken from the vertex to 2 cm below the sternoclavicular joint at a slice thickness of 3 mm. The target volumes were delineated slice-by-slice on treatment planning CT scans using an individualized delineation protocol according to the International Commission on Radiation Units and Measurements reports 50 and 62. All targets were treated using the simultaneous integrated boost technique. The biological equivalent dose (BED) for the ENLNs was calculated using the following formula:

$${\text{BED}} = nd \times \left( { 1 + d/\left[ {\alpha /\beta } \right]} \right),$$

with *nd* represents the total radiation dose, *d* represents fraction dose, *α*/*β* represents the biological effect of tumor cells to radiation (the value for tumor tissues is 10) [7].

#### Chemotherapy

According to our institutional guidelines, prior to commencing the treatment, we recommended radiotherapy alone for stage I disease, CCRT for stage II disease, and CCRT alone or in combination with neoadjuvant/adjuvant chemotherapy for stage III to IVA-B disease. Neoadjuvant or adjuvant chemotherapy regimen comprised cisplatin (80 mg/m^2^ on day 1) with 5-fluorouracil (750 mg/m^2^ on days 1–5) or cisplatin (75 mg/m^2^ on day 1) with docetaxel (75 mg/m^2^ on day 1) every 3 weeks for two or three cycles. Concurrent chemotherapy regimen comprised cisplatin (30–40 mg/m^2^ on day 1) administered every week or cisplatin (80–100 mg/m^2^ on day 1) on weeks 1, 4, and 7 of radiotherapy.

### Follow-up and statistical analysis

Patient follow-up was measured from the first day of therapy to the last visit (August 8, 2016) or death. The patients were examined at least every 3 months during the first 2 years and every 6 months thereafter until death. The end-points (time to first defining event) were regional relapse-free survival (RRFS, time to regional node recurrence), disease-free survival (DFS, time to disease recurrence or death), overall survival (OS, time to death from any cause), and distant metastasis-free survival (DMFS, time to distant metastasis); local relapse-free survival (LRFS) was not assessed in the present study. RRFS was the primary end-point of the present study.

Receiver operating characteristic (ROC) curves were used to calculate the cut-off value of the ENLN diameter for the prediction of DFS. The cut-off value of serum lactate dehydrogenase (LDH) level was determined as previously described [8, 9]. The Chi square test was used to compare clinical characteristics. Life-table estimation was performed using the Kaplan–Meier method and log-rank test. The multivariate Cox proportional hazards model was used to estimate the hazard ratio (HR) and 95% confidence interval (CI). All statistical tests were two-sided; *P* < 0.05 was considered significant. STATA statistical package (STATA 12; StataCorp LP, College Station, Texas, USA) was used for all analyses.

## Results

### ENLN diameter

**Fig. 1:**
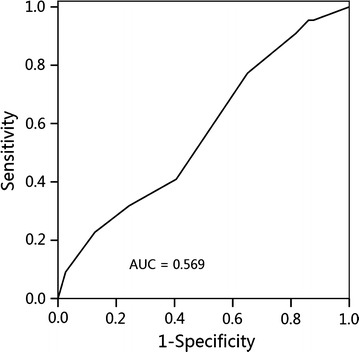
Receiver operating characteristic curve analysis of the short diameter of the largest enlarged neck lymph node (ENLN) in patients with N0-category nasopharyngeal carcinoma (NPC) for the prediction of disease-free survival. *AUC* area under the curve

A total of 1811 patients were diagnosed with non-disseminated NPC at Sun Yat-sen University Cancer Center between November 2009 and February 2012, and 251 (13.9%) were selected in the present study. The median diameter of the largest ENLN for the 251 patients was 6 mm (range, 3–10 mm). Based on an analysis of the ROC curve (Fig. 1), the cut-off value of the ENLN diameter for the prediction of DFS was 5.5 mm (area under the curve [AUC], 0.569; sensitivity, 0.773; specificity, 0.349). Therefore, patients were divided into small and large ENLN groups using this cut-off value: 85 (33.9%) patients had ENLN <5.5 mm, and 166 (66.1%) patients had ENLN ≥5.5 mm in the short diameter.

### Baseline characteristics and radiation dose

Of the 251 patients, 194 (77.3%) were males, and 57 (22.7%) were females, with a male-to-female ratio of 3.4:1. The median age was 46 years (range, 19–77 years). The small and large ENLN groups were similar in terms of host factors, tumor stage, and treatment (all *P* > 0.05) (Table 1).

**Table 1:** Baseline characteristics of the 251 patients with N0-category nasopharyngeal carcinoma (NPC) and enlarged neck lymph nodes (ENLNs)

Characteristic	Total (cases)	Small ENLN group [cases (%)]	Large ENLN group [cases (%)]	*P* value^a^
Gender				0.923
Male	194	66 (77.6)	128 (77.1)	
Female	57	19 (22.4)	38 (22.9)	
Age (years)				0.661
<50	167	55 (64.7)	112 (67.5)	
≥50	84	30 (35.3)	54 (32.5)	
Smoking				0.935
Yes	73	25 (29.4)	48 (28.9)	
No	178	60 (70.6)	118 (71.1)	
Drinking				0.836
Yes	25	8 (9.4)	17 (10.2)	
No	226	77 (90.6)	149 (89.8)	
LDH (U/L)				0.973
<245	242	82 (96.5)	160 (96.4)	
≥245	9	3 (3.5)	6 (3.6)	
Family history of cancer				0.332
Yes	87	26 (30.6)	61 (36.7)	
No	164	59 (69.4)	105 (63.2)	
BED (Gy)				0.821
<72	91	30 (35.3)	61 (36.7)	
≥72	160	55 (64.7)	105 (63.3)	
Chemotherapy				0.222
Yes	152	47 (55.3)	105 (63.3)	
No	99	38 (44.7)	61 (36.7)	
T category^b^				0.270
T1	80	27 (31.8)	53 (31.9)	
T2	54	24 (28.2)	30 (18.1)	
T3	87	25 (29.4)	62 (37.3)	
T4	30	9 (10.6)	21 (12.7)	

The patients were divided into small and large ENLN groups using the cut-off value (5.5 mm) of the short diameter of the largest ENLN for the prediction of DFS

*LDH* lactate dehydrogenase, *BED* biological equivalent dose

^a^ *P* values were calculated using the Chi square test or Fisher’s exact test if indicated

^b^According to the 7th edition of the American Joint Committee on Cancer/Union for International Cancer Control (AJCC/UICC) staging system

The prescribed doses were 66–72 Gy at 2.12–2.43 Gy/fraction to the planning target volume (PTV) of the primary gross tumor volume (GTVnx), 50–68 Gy at 1.67–2.27 Gy/fraction to the PTV of the ENLN (GTVnd), 60–63 Gy to the PTV of the high-risk clinical target volume (CTV1), and 50–56 Gy to the PTV of the low-risk clinical target volume (CTV2). The median BED to GTVnd was 72.0 Gy (range, 58.3–83.4 Gy) for the entire cohort, 72.0 Gy (range, 58.3–83.4 Gy) for the small ENLN group, and 72.0 Gy (range, 58.3–82.0 Gy) for the large ENLN group. The BED was not significantly different between the small and large ENLN groups (*t* test, *P* = 0.418).

### Treatment failure patterns

Up to the last follow-up (August 8, 2015), 16 (6.4%) patients were lost to follow-up. The median follow-up duration was 49.3 months (range, 11.0–67.7 months) for the entire cohort and 49.8 months (range, 11.3–67.7 months) for the surviving patients. In total, 10 (4.0%) patients developed local recurrence, 2 (0.8%) experienced regional recurrence, and 12 (4.8%) suffered distant metastasis. Moreover, 12 cancer-related deaths were observed. The failure patterns in the two groups are summarized in Table 2.

**Table 2:** Failure patterns in the small and large ENLN groups of NPC patients

Failure pattern	Small ENLN group [cases (%)]	Large ENLN group [cases (%)]	*P* value^a^
Local recurrence	0 (0)	10 (6.0)	0.049
Regional recurrence	2 (2.3)	0 (0)	0.217
Total locoregional failure	2 (2.3)	10 (6.0)	0.328
Distant metastasis	2 (2.3)	10 (6.0)	0.328
Total death	3 (3.5)	9 (5.4)	0.725
Total failure	5 (5.9)^b^	17 (10.2)^c^	0.248

^a^ *P* values were calculated using Chi square test or Fisher exact test if indicated

^b^One patient died without disease recurrence and distant metastasis

^c^Three patients experienced both local and distant failure

### Univariate and multivariate analysis

**Fig. 2:**
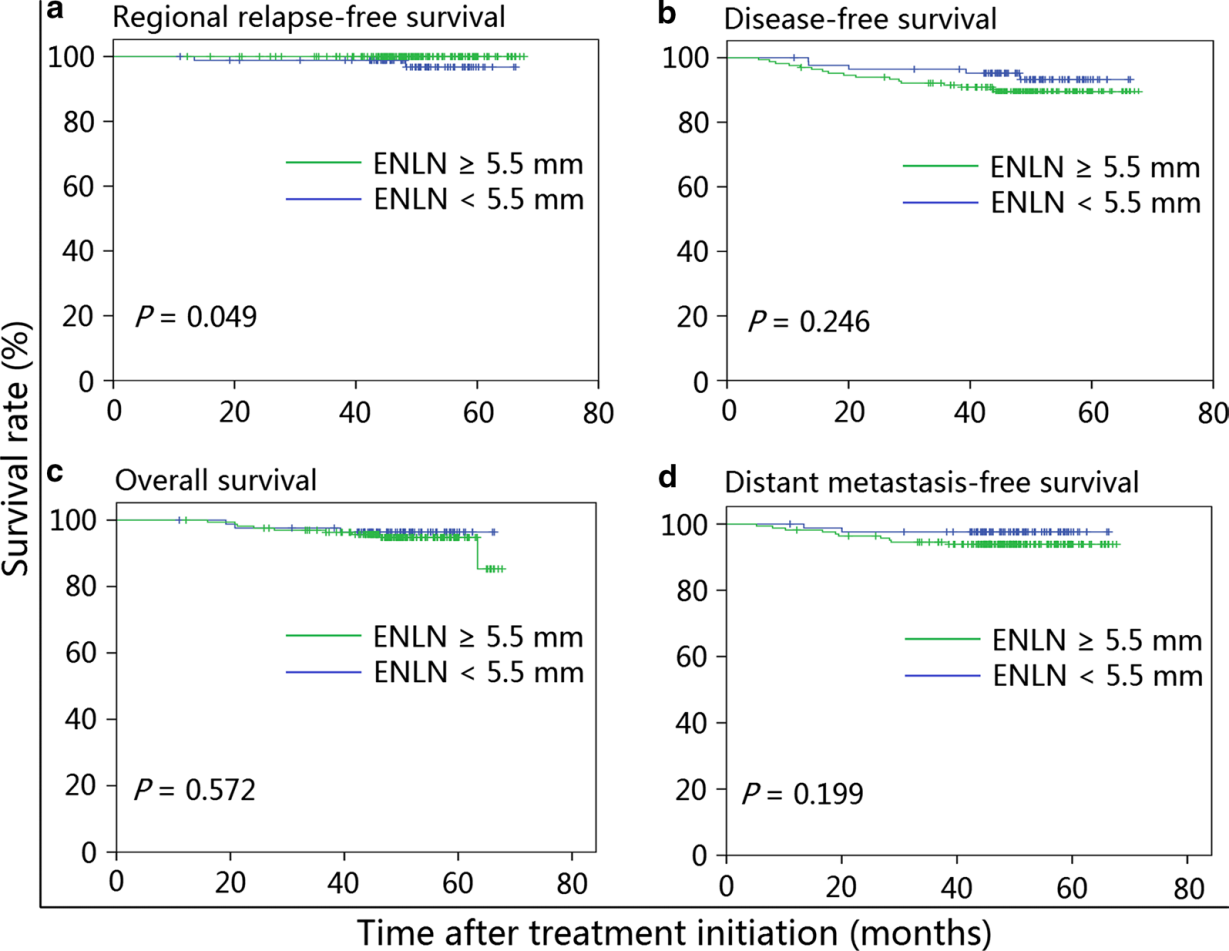
Kaplan–Meier survival curves of patients with N0-category NPC and ENLN in the small ENLN (<5.5 mm) and large ENLN (≥5.5 mm) groups. **a** Regional relapse-free survival; **b** disease-free survival; **c** overall survival; **d** distant metastasis-free survival. *NPC* nasopharyngeal carcinoma, *ENLN* small neck lymph node

For the entire cohort, the estimated 4-year RRFS, DFS, OS, and DMFS rates were 99.6, 91.4, 95.3, and 95.2%, respectively. For patients in the large and small ENLN groups, except for the estimated 4-year RRFS rate (100% vs. 98.8%, *P* = 0.049; Fig. 2a), the estimated 4-year DFS (89.4% vs. 95.2%, *P* = 0.246; Fig. 2b), OS (94.8% vs. 96.4%, *P* = 0.572; Fig. 2c), and DMFS rates (93.9% vs. 97.6%, *P* = 0.199; Fig. 2d) were comparable. Moreover, the 4-year RRFS (100% vs. 99.4%, *P* = 0.278), DFS (95.4% vs. 89.1%, *P* = 0.062), OS (97.7% vs. 94.0%, *P* = 0.311), and DMFS rates (97.8% vs. 93.7%, *P* = 0.147) were also comparable between patients receiving BED < and ≥72 Gy.

Multivariate analysis was performed to adjust for various factors, including gender, age, smoking, drinking, serum LDH level, family history of cancer, BED, chemotherapy, T category, and ENLN diameter. Both ENLN diameter and BED were not established as independent prognostic factors for RRFS, DFS, OS, or DMFS (all *P* > 0.05). Moreover, no significant prognostic factor was identified for RRFS (Table 3).

**Table 3:** Multivariate analysis of the prognosis of 251 patients with N0-category NPC and ENLNs using an adjusted Cox proportional hazards model

Variable	RRFS			DFS			OS			DMFS		
	HR	95% CI	*P* value	HR	95% CI	*P* value	HR	95% CI	*P* value	HR	95% CI	*P* value
Gender (male vs. female)	<0.001	–	0.666	1.548	0.530–4.525	0.424	1.089	0.223–5.307	0.916	1.562	0.340–7.182	0.567
Age (≥50 vs. <50 years)	<0.001	–	0.583	2.318	0.998–5.386	0.051	7.955	1.916–33.026	0.004	2.821	0.892–8.919	0.077
Smoking (yes vs. no)	0.009	–	0.841	1.680	0.686–4.116	0.257	2.608	0.713–9.536	0.147	2.393	0.759–7.544	0.136
Drinking (yes vs. no)	0.001	–	0.870	0.350	0.047–2.632	0.308	<0.001	–	0.980	<0.001	–	0.985
Serum LDH level (<245 vs. ≥245 U/L)	<0.001	–	0.857	4.689	1.370–16.050	0.014	15.445	3.224–73.992	0.001	5.794	1.258–26.693	0.024
Family history of cancer (yes vs. no)	132.973	–	0.585	0.764	0.302–1.933	0.570	0.199	0.034–1.172	0.074	0.602	0.160–2.270	0.454
BED (<72 vs. ≥72 Gy)	107,046.092	–	0.485	2.438	0.882–7.226	0.108	3.097	0.697–13.754	0.137	2.741	0.60–12.526	0.193
Chemotherapy (yes vs. no)	<0.001	–	0.473	1.123	0.409–3.084	0.821	1.132	0.235–5.465	0.877	0.924	0.228–3.752	0.912
T category (T1–2 vs. T3–4)	<0.001	–	0.539	1.204	0.510–2.842	0.672	2.984	0.649–13.720	0.160	1.497	0.468–4.794	0.497
ENLN diameter (<5.5 vs. ≥5.5 mm)	<0.001	–	0.498	1.914	0.704–5.200	0.203	1.288	0.308–5.392	0.729	2.579	0.556–11.969	0.226

*RRFS* regional relapse-free survival, *DFS* disease-free survival, *OS* overall survival, *DMFS* distant metastases-free survival, *HR* hazard ratio, *CI* confidence interval, *LDH* lactate dehydrogenase, *BED* biological equivalent dose

– No confidence interval

### Subgroup analysis

**Fig. 3:**
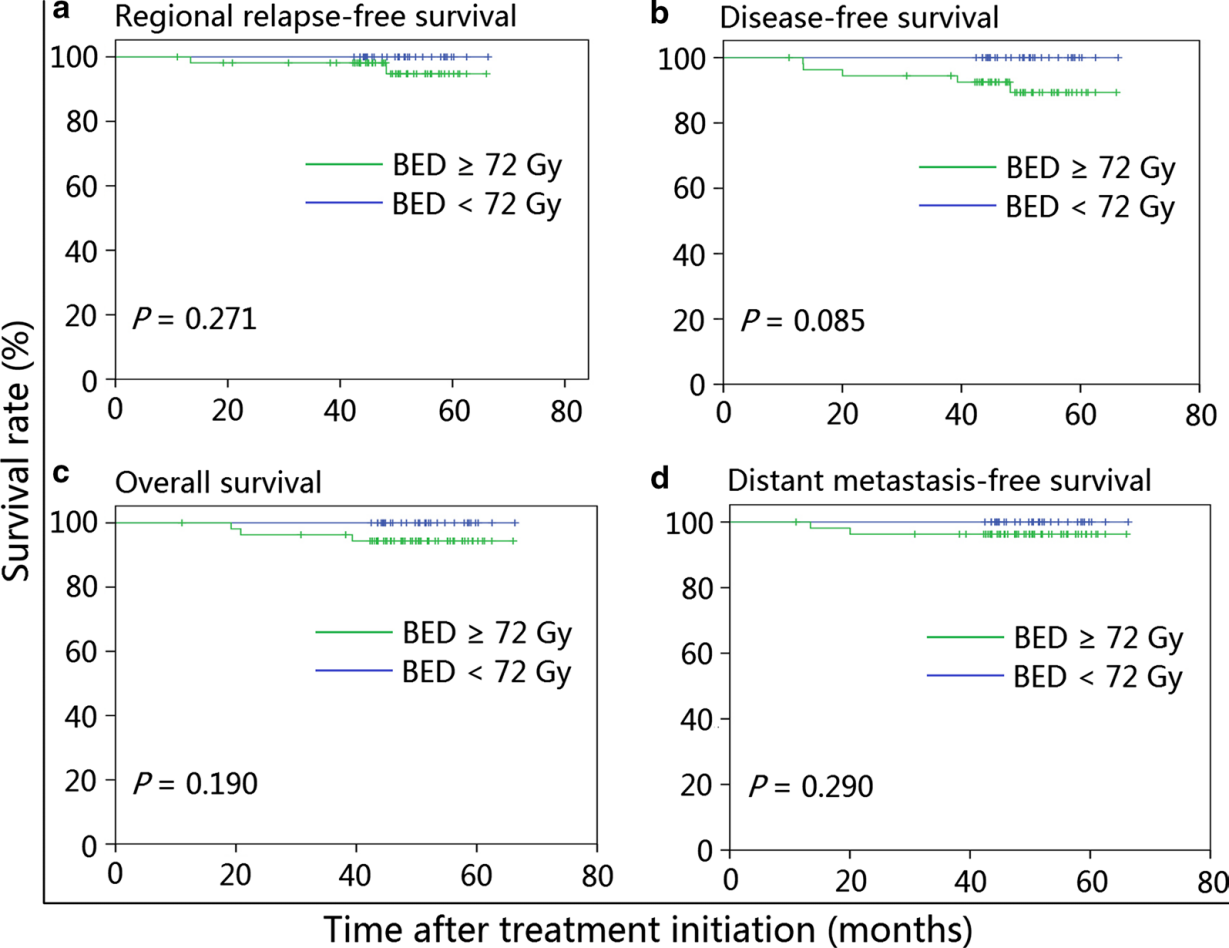
Kaplan–Meier survival curves of N0-category NPC patients in the small ENLN group stratified by a biological equivalent dose (BED) < or ≥72 Gy. **a** Regional relapse-free survival; **b** disease-free survival; **c** overall survival; **d** distant metastasis-free survival. *NPC* nasopharyngeal carcinoma, *ENLN* enlarged neck lymph node

**Fig. 4:**
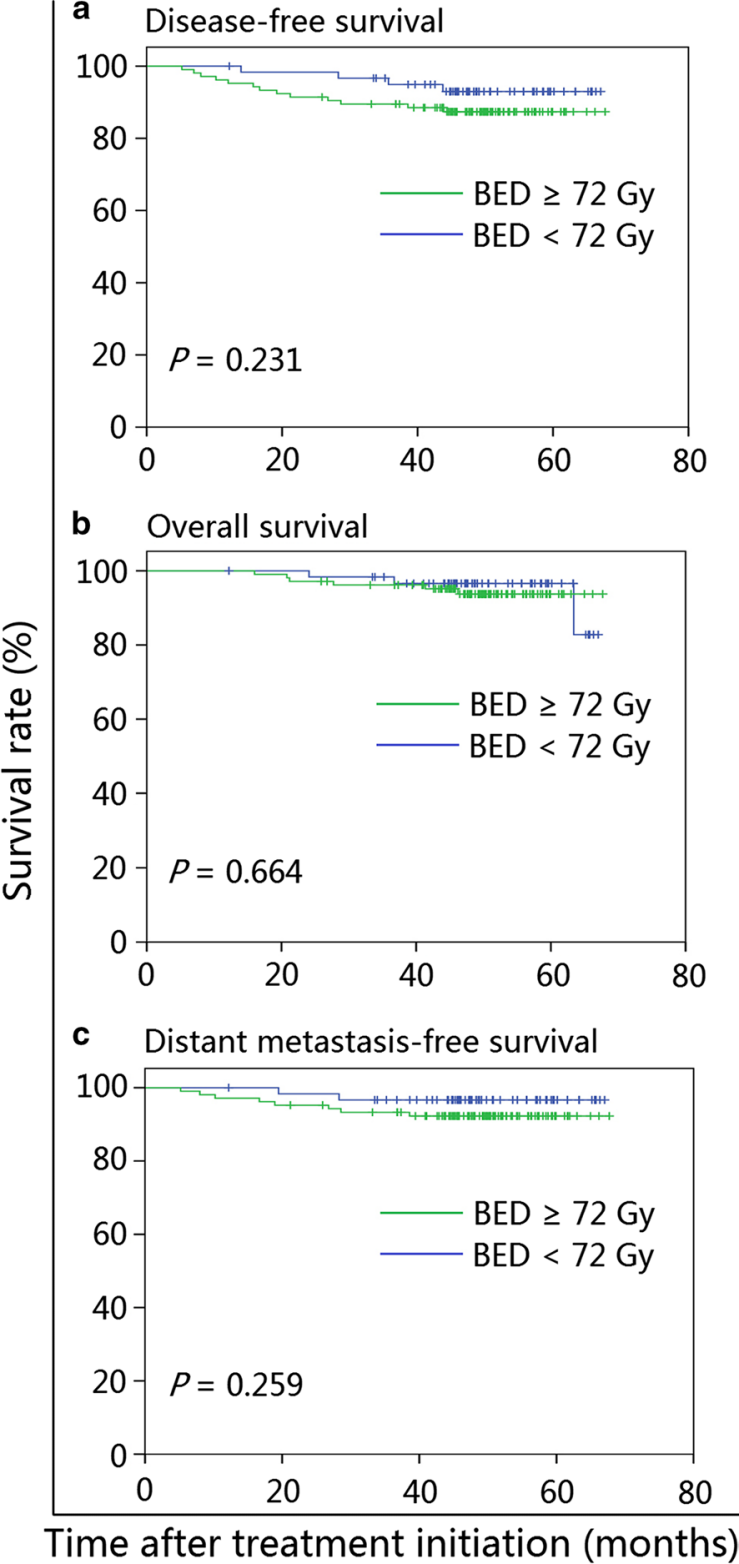
Kaplan–Meier curves for prognostication by ENLN ≥5.5 mm stratified by a BED < or ≥72 Gy: (**a**) disease-free survival; (**b**) overall survival; (**c**) distant metastasis-free survival

Furthermore, we conducted a stratified analysis to characterize the prognostic value of different BED in the small and large ENLN groups. The estimated 4-year RRFS, DFS, OS, and DMFS rates were comparable between patients receiving a BED ≥72 Gy and <72 Gy in both the small ENLN group (Fig. 3) and the large ENLN group (Fig. 4).

## Discussion

In the present study, we found that ENLN diameter was not an independent prognostic factor for patients with N0-category NPC and ENLN and that a BED ≥72 Gy to the ENLNs was not associated with significantly prolonged RRFS, DFS, OS, or DMFS in both small and large ENLN groups. Moreover, consistent with previous results [8, 9], LDH was the most important predictor of DFS, OS, and DMFS.

Previous studies reported that 13.6% [4] and 15% [10] of patients with NPC had no lymph node metastasis, similar to the results of the present study (13.9%). In the cohort examined in the present study, tumor stage and treatment-related factors were well balanced between the small and large ENLN groups, and univariate analysis revealed that the 4-year RRFS rate was significantly higher in the small ENLN group than in the large ENLN group (*P* = 0.049). However, after adjusting for various factors, multivariate analysis did not establish ENLN diameter as an independent predictor of RRFS. There are three potential reasons that may explain this result. First, the ENLNs are pathologically negative and have no prognostic impact. Second, the radiation dose received by the ENLNs may be sufficient to provide a radical treatment effect and, therefore, result in a satisfactory prognosis. Third, the relatively small sample size of each group may have resulted in a low statistical power. Indeed, reflecting the excellent regional control rates, no independent predictor was identified for RRFS among this cohort of patients with N0-category NPC.

Notably, the retropharyngeal small LNs were not analyzed in the present study because they are adjacent to the primary tumor and are often treated as part of the primary tumor volume, even if they do not fit the diagnostic criteria for positive LNs in clinical practice. Therefore, almost all negative retropharyngeal small LNs received a radical radiation dose, which would have affected the results of the present study if these nodes were included in the analysis. A comparison of the failure patterns between groups revealed that the large ENLN group had a significantly higher rate of local failure than the small ENLN group. However, local relapse-free survival (LRFS) was not assessed as an endpoint in the present study because the ENLNs would not affect the local control. Therefore, we did not perform univariate analysis of the LRFS for the two groups.

Many studies have solely focused on the feasibility of omitting irradiation to the lower neck in N0-category NPC [11–14]. However, no studies have investigated the optimal radiation dose for the ENLNs. Notably, the doses prescribed to the ENLNs greatly varied in the present study, and the highest dose was 68 Gy—the radical radiation dose for positive LNs. This finding reflects clinicians’ concern that the ENLNs may be positive and could adversely affect prognosis, and should, therefore, receive a radical radiation dose. Subgroup analysis was conducted to further investigate the prognostic value of the BED for patients in different ENLN groups to establish the optimal BED for the ENLNs. We found that patients receiving a BED ≥72 Gy had a similar prognosis to patients receiving a BED <72 Gy in both the small and large ENLN groups, indicating that a total BED of 72 Gy may be sufficient for the ENLNs. Moreover, Li et al. [14] also revealed that the administration of 60 Gy in 30 fractions (BED = 72 Gy) to the suspicious node area did not lead to cervical node relapse, further supporting the results obtained in the present study. This finding should be of great importance to clinicians when devising individualized treatment strategies.

Based on the results of the present study, we provide credible evidence to reassure clinicians that the ENLNs do not negatively affect the prognosis of patients with N0-category NPC in the era of IMRT if a total BED of 72 Gy is achieved. The BED for the treatment of the ENLNs should not exceed 72 Gy, as excessive radiation doses will cause acute toxicities, such as radiation-related dermatitis, and severe long-term toxicities, such as neck muscle atrophy [15].

Several limitations exist in the present study. First, the follow-up duration may be insufficient to observe a significant difference of OS since patients with N0-category disease had excellent prognoses. Therefore, we set RRFS as the first endpoint to address this issue because a median follow-up of 49.3 months was sufficient to determine the difference in RRFS. Second, the assessment of the ENLNs could only be performed reliably using MRI because, unlike other head and neck cancers, NPC is typically treated without pathologic analysis of the ENLNs. Third, the sample size and number of regional failure events in each group were relatively small. Moreover, due to incomplete medical records, several other important prognostic factors, including plasma Epstein-Barr virus DNA [16–22] and primary tumor volume [23, 24], could not be assessed in the present study. Therefore, further prospective clinical studies with larger cohorts that include additional prognostic factors are warranted to confirm the outcomes of the present study.

## Conclusions

The present retrospective study provides evidence suggesting the treatment of patients with N0-category NPC and ENLNs. A total BED of 72 Gy to the ENLNs is considerably sufficient to provide a clinical benefit, and excessive radiation doses could be avoided to reduce acute and long-term toxicities. However, prospective randomized studies are warranted to validate the findings obtained in the present study.
